# From asymptomatic adult patient to cardiopulmonary resuscitation – treatment of ALCAPA with total arterial myocardial revascularisation and mitral valve repair

**DOI:** 10.1186/s13019-024-02906-5

**Published:** 2024-06-25

**Authors:** Mustafa Al-Obaidi, Florian Hecker, Thomas Walther, Tomas Holubec

**Affiliations:** Department of Cardiovascular Surgery, Heart Centre, Goethe University and University Hospital Frankfurt, Frankfurt/Main, Germany

**Keywords:** Anomalous origin of the left coronary artery from the pulmonary artery (ALCAPA), Coronary artery bypass grafting, Mitral valve repair

## Abstract

**Background:**

Anomalous left coronary artery originating from the pulmonary artery (ALCAPA), is a unique congenital anomaly, comprising only 0.24–0.46% of all congenital cardiac anomalies. Late presentations, ranging from asymptomatic cases to sudden cardiac arrest, are exceptionally rare. This unique case highlights the complexity of managing ALCAPA in adulthood and underscores the necessity of a comprehensive surgical approach addressing both coronary and valvular issues.

**Presentation:**

A 34-year-old female patient, who had been diagnosed with ALPACA in her early childhood, suffered sudden cardiac arrest at work, followed by out-of-hospital resuscitation. The patient had been followed-up regularly until adolescence, however, she had refused surgery. In the diagnostic work-up following successful resuscitation severe mitral valve regurgitation was additionally revealed. Subsequently, the patient underwent surgery, involving coronary artery bypass grafting (CABG), using bilateral internal mammary arteries, and mitral valve repair, with an excellent postsurgical result. At 16-month follow-up, the patient was asymptomatic and quality of life had significantly improved.

**Conclusion:**

This rare case initially presented as silent myocardial ischemia, resulting in reduced left ventricular function and secondary mitral incompetence. Surgical treatment of ALCAPA in adults poses greater challenges and a higher risk than in children. CABG procedure offers an excellent prognostic therapeutic strategy, since this procedure is a routine in adult cardiac surgery.

## Background

Anomalous left coronary artery originating from the pulmonary artery (ALCAPA) was first described by Abbott in 1908 [1]. Further clinical description followed in 1933 by Bland, White, and Garland. Therefore, ALCAPA is also referred to as Bland-White-Garland syndrome [2]. Without surgical correction, ALCAPA has a mortality rate of approximately 90% in the first year of life. Ten percent of patients survive due to the development of numerous intercoronary collaterals, ensuring myocardial perfusion. Of these patients, approximately 90% suffer sudden cardiac death at an average age of 35 years [3].Thus, most adults with ALCAPA die without having been diagnosed the condition. Therefore, adults with ALCAPA present quite rarely in clinical practice, with clinical presentation ranging from asymptomatic to sudden cardiac arrest.

## Embryology and pathophysiology

ALCAPA arises due to either a defect in septation of the conotruncus into aorta and pulmonary artery or malposition of the coronary buds [4]. Prenatally, a patent ductus arteriosus provides relatively equivalent pressure in the main pulmonary artery and aorta, and therefore a relatively equivalent oxygenation and perfusion of the myocardium. After birth, closure of the ductus arteriosus occurs. Thus, the pressure in the main pulmonary artery decreases, resulting in a reverse blood flow from the left coronary artery to the pulmonary artery. This steal phenomenon leads to inadequate perfusion of the left ventricular myocardium from the anomalous left coronary artery. In the case of poor collateral circulation, myocardial ischemia develops, progressing to infarction, dilatation of the left ventricle, and thus congestive heart failure, mitral valve regurgitation, arrhythmia and sudden death. Patients with an adequate collateral supply from the right coronary artery may survive to adulthood [5].

## Case presentation

**Fig. 1:**
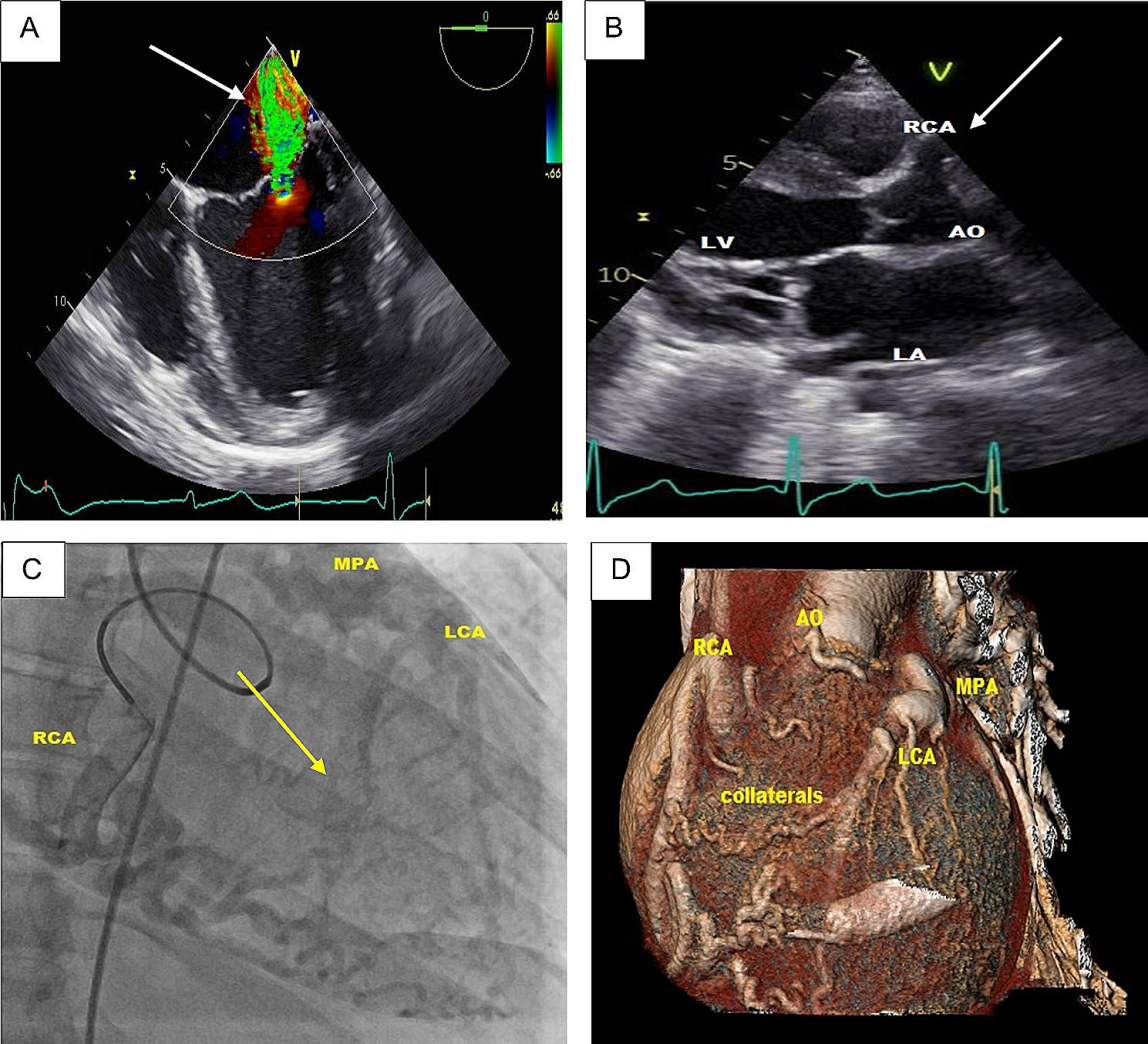
Preoperative diagnostic images. **A**) Transthoracic echocardiogram showing regurgitation jet (arrow) of the mitral valve. **B**) Transthoracic echocardiogram (parasternal view) showing the dilated right coronary artery (RCA) ostium (arrow). **C**) Coronary angiogram demonstrating a tortuous dilated RCA giving intercoronary collaterals (arrow) to the left coronary artery (LCA) which drains into the main pulmonary artery (MPA). **D**) CT scan with three-dimensional reconstruction demonstrating ALCAPA syndrome. AO: Aorta, LA: left atrium, LV: left ventricle, RV: right ventricle, RCA: right coronary artery, LCA: left coronary artery, MPA: main pulmonary artery, CT: computer tomography

A 34-year-old female suffered a sudden witnessed collapse at work. Out-of-hospital cardio-pulmonary resuscitation was initiated and after 8 min and cardiac defibrillation spontaneous circulation was re-established. The patient was admitted to the intensive care unit and extubated the next day without neurological complications. Transthoracic echocardiography (TTE) revealed a dilated left ventricle with globally reduced ejection fraction of 40% due to ischemia, leading to fibrosis and malfunction of papillary muscles causing a tethering of the mitral valve leaflets. This resulted in secondary moderate to severe mitral valve regurgitation. In addition, a mitral annular dilatation contributed further to the severity of the mitral valve insufficiency (Fig. 1A). Furthermore, the right coronary artery ostium was found to be dilated (Fig. 1B). The coronary angiogram and CT scan confirmed the diagnosis of ALCAPA syndrome (Fig. 1C and D). Following the discussion of the case in our interdisciplinary heart team, the patient was scheduled for urgent surgical therapy.

## Surgical procedure

**Fig. 2:**
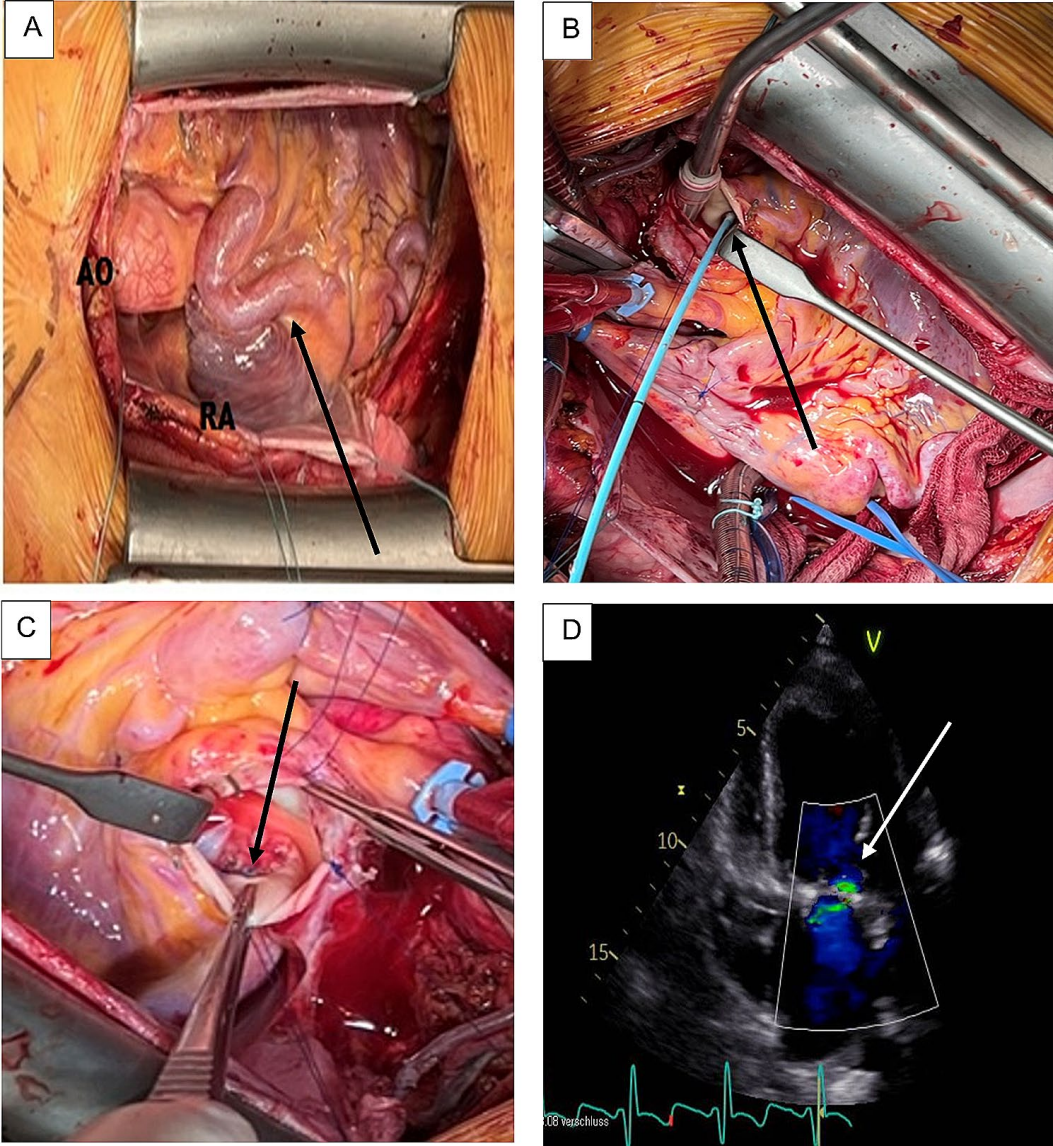
Intraoperative and postoperative images. **A**) Intraoperative image demonstrating tortuous, dilated dominant RCA (arrow). **B**) Intraoperative photograph showing the ALCAPA ostium (arrow), the tip of the 4 F Fogarty® catheter (Edwards Lifesciences LLC; Irvine/CA, USA) is inserted in the LCA ostium. **C**) Occluded ALCAPA ostium using autologous pericardial patch (arrow). **D**) Postoperative transthoracic echocardiogram showing mild residual mitral valve regurgitation (arrow). AO: Aorta, RA: right atrium, RCA: right coronary artery, LCA: left coronary artery

After median sternotomy, both internal mammary arteries (IMAs) were harvested, and the pericardium was opened. The dilated right coronary artery was observed (Fig. 2A). After establishment of cardio-pulmonary bypass, the aorta was cross-clamped and antegrade cold cardioplegia (modified Del Nido solution) was administered. The establishment of cardiac arrest was difficult due to steal phenomenon from the right to left coronary artery via collaterals, therefore the main pulmonary artery was dissected and the ostium of the ALCAPA identified postero-laterally. Consequently, the ostium of ALCAPA was blocked using a 4 F Fogarty® catheter (Edwards Lifesciences LLC; Irvine/CA, USA; Fig. 2B). Additional cardioplegic solution was administered via this catheter and sufficient cardiac arrest was accomplished. The left and right IMA was anastomosed to the obtuse marginal branch of the circumflex coronary artery and to the left anterior descending artery, respectively. The ALCAPA ostium was then occluded using an autologous pericardial patch treated with 0.6% Glutaraldehyde (Fig. 2C). Inspection of the mitral valve revealed a thickening of the posterior mitral leaflet with annular dilatation. Mitral annuloplasty was carried out using a 34 mm ETLogix® annuloplasty-ring (Edwards Lifesciences LLC; Irvine/CA, USA). The intraoperative course was uneventful, and the patient had an uncomplicated further postoperative course with early extubation and fast recovery. The patient was discharged on the seventh postoperative day. TTE performed at two months follow-up revealed improved contractility with increased ejection fraction of 45%, with a mild residual mitral valve regurgitation (Fig. 2D). The last follow-up at 16 months showed an excellent postoperative result with significant improvement in quality of life. The TTE revealed further increase of the left ventricular ejection fraction (up to 50%) with a mild to moderate mitral regurgitation.

## Discussion

In the majority of patients, ALCAPA syndrome is a lethal congenital anomaly posing a substantial risk of myocardial ischemia, arrhythmia, and sudden death. While conservative medical therapy is considered for elderly patients with mild perfusion impairment [6], surgery remains the standard, essential therapy to prevent cardiac ischemia and sudden cardiac death in ALCAPA syndrome. The surgical approach encompasses two primary goals: firstly, restoring a dual-coronary artery system for sufficient myocardial perfusion, preventing arrhythmia and sudden death; secondly, ensuring long-term graft patency. Various surgical methods have been reported, all of which have their pitfalls, including: (1) simple occlusion of the left main coronary artery origin [7], which proved insufficient in preventing sudden death, as the patient is left with only one coronary artery; (2) direct re-implantation of the left coronary artery into the aorta [8], which is challenging in adults due to unfavourable anatomy related to the limited length and reduced elasticity of the left coronary artery, collaterals surrounding the coronary artery origin, and higher bleeding tendency [9]; (3) saphenous vein graft between the origin of the left coronary artery and the aorta [10], not recommended due to short patency of the graft along with the reported risk of occlusion; (4) Takeuchi repair involving tunnelling the anomalous ostium of the left coronary artery across the pulmonary artery to the aorta [11], may be associated with coronary conduit stenosis, pulmonary artery stenosis, and aortic stenosis or regurgitation; and (5) CABG procedure using the IMA as a graft along with the occlusion of the ALCAPA ostium [7]. CABG has been found to be a convenient procedure in adults with ALCAPA, offering simplicity, avoidance of aortic root manipulation, and a reliable long-term patency of the IMA graft. Although the competitive flow from the native coronary artery can present a considerable risk of early graft failure, it has been reported that the IMA graft can adapt to the sustained competitive blood flow conditions [12]. The patency of the IMA after CABG procedure in patients with atherosclerotic coronary artery disease increases with the severity of the native coronary artery stenosis. At 15-year follow-up, IMA patency was 93% when stenosis of the left anterior descending artery was 50% and 98% when stenosis was > 90%, respectively [13].

Applying bilateral IMA safeguards against coronary artery ischemia and overcomes the steal phenomenon, solidifying its status as a comprehensive and effective surgical strategy for ALCAPA syndrome in adults.

## Conclusion

ALCAPA is a rare and lethal condition necessitating an early surgical correction to re-establish a dual-coronary system, which is defined as the gold standard therapy. In an array of surgical techniques CABG is a safe and practical procedure, that can be performed in patients with ALCAPA with good early results. Taking into account the use of arterial grafts and ligation of the native coronary artery, as this may enhance the long-term patency of the graft and inhibits the competitive flow, thus avoiding graft atrophy and failure.

Additionally, a close and long-term follow-up is an essential postoperative part of the treatment of ALCAPA with bypass surgery.

As a result, bypass surgery should be viewed as one of the choices in the treatment of ALCAPA in adults, complemented by mitral valve repair, if necessary. This comprehensive approach can be considered a safe and reliable therapy for both coronary arteries and valve dysfunction, with early intervention being of great importance.

## Data Availability

No datasets were generated or analysed during the current study.

## Abbreviations

ALCAPA: Anomalous origin of the left coronary artery from the pulmonary artery
CABG: Coronary artery bypass grafting
TTE: Transthoracic echocardiography
CT: Computed tomography
IMA: Internal mammary artery
